# CRISPR/Cas9-edited *NSG* mice as PDX models of human leukemia to address the role of niche-derived SPARC

**DOI:** 10.1038/leu.2017.346

**Published:** 2017-12-06

**Authors:** I Tirado-Gonzalez, E Czlonka, A Nevmerzhitskaya, D Soetopo, E Bergonzani, A Mahmoud, A Contreras, I Jeremias, U Platzbecker, J P Bourquin, U Kloz, F Van der Hoeven, H Medyouf

**Affiliations:** 1 Georg-Speyer-Haus, Institute for Tumor Biology and Experimental Therapy, Frankfurt, Germany; 2 Department of Apoptosis in Hematopoietic Stem Cells, Helmholtz Center Münich, German Center for Environmental Health (HMGU), Munich, Germany; 3 German Cancer Consortium, DKTK Partner Site Munich, Heidelberg, Germany; 4 University Hospital Carl Gustav Carus, Technical University Dresden, Dresden, Germany; 5 German Cancer Consortium, DKTK Partner Site Dresden, Heidelberg, Germany; 6 Division of Pediatric Oncology, University Children’s Hospital, Zurich, Switzerland; 7 Transgenic Service, German Cancer Research Center, Heidelberg, Germany; 8 German Cancer Consortium, DKTK Partner Site Frankfurt/Mainz, Heidelberg, Germany; 9 Department of Hematology and Oncology, University Hospital Mannheim, Medical Faculty, University of Heidelberg, Mannheim, Germany

**Keywords:** Cancer, Biotechnology, CRISPR-Cas9 genome editing, Cancer models, Glycoproteins, Leukaemia

Patient-derived xenografts (PDX) represent invaluable tools to study the biology of human cancers *in vivo*. The severely immuno-compromised NOD *Prkdc*^*scid*^ *Il2rg*^*−/−*^ (NSG) mouse strain is the most commonly used strain, in particular for hematologic malignancies. However, the lack of genetically modified (GM) NSG mice has greatly hampered functional studies in PDX models, in particular those that address the role of the microenvironment. The tumor microenvironment is a key player in cancer progression and a major contributor to therapeutic resistance.

A handful of studies have reported the generation of GM NSG mice through a time-consuming backcross of GM C57BL/6 mice onto an NSG background.^1, 2, 3, 4^ Here we describe a strategy that uses CRISPR (Clustered Regularly Interspaced Short Palindromic Repeats)/Cas9 (CRISPR associated protein 9) technology to efficiently generate GM NSG mice to specifically interrogate the role of niche-derived SPARC (Secreted Protein Acidic Rich in Cysteine) *in vivo* in different human leukemic contexts.

SPARC is a multifaceted matricellular protein that regulates key physiological processes by modulating cell–cell and cell–matrix interactions. Moreover, SPARC is shown to be differentially expressed in various tumors and proposed to modulate cancer cell activity.^5^ In leukemic contexts, SPARC is proposed to exert opposing effects depending on the leukemia type or even the specific subtype under study.^6, 7^ However, no study has so far investigated the role of niche-derived SPARC in human leukemia, in an *in vivo* setting.

In recent years, the use of targeted nucleases has revolutionized the field of genome engineering. In particular, CRISPRs and the Cas9 endonuclease system has proven to be by far the most versatile system. Cas9-induced double-strand breaks (DSBs) are rapidly resolved by DNA-dependent protein kinase (DNA-PK)-dependent non-homologous end joining (NHEJ), a repair mechanism that generates InDels and often results in functional inactivation of the targeted gene. Alternatively, precise editing can be achieved through the use of a donor template DNA homologous to the sequences flanking the DSB through a homology-directed repair (HDR) mechanism that is independent of DNA-PK.^8^ In wild-type cells, HDR is largely outcompeted by the fast acting NHEJ. However, because of the *Prkdc*^*scid*^ mutation, NSG mice do not express DNA-PK and as such are impaired in their ability to efficiently resolve DSBs by NHEJ. We hypothesized that this deficiency would allow for efficient HDR, a fact that could be exploited to achieve precise genome editing in NSG background.

NSG zygotes were microinjected in the cytoplasm with Cas9, guide RNA (gRNA) and a template single-stranded DNA (ssDNA) (Supplementary Figure 1A and Supplementary Methods online), as previously described.^9^ Guide RNAs targeting exon 4 (gRNA#1) or exon 2 (gRNA#2) (Supplementary Figure1B) were initially tested for their ability to induce on target editing by transient transfection in NHEJ competent MS5 murine stromal cells. Using the surveyor assay, we observed efficient genome editing with both gRNAs. Although gRNA#2 was more efficient (Supplementary Figure 1C), we elected to use gRNA#1 because of the reduced number of predicted off-targets (Supplementary Table 1). To achieve a functional knockout in NSG mice, we used gRNA1 that targets an early-translated exon of the *Sparc* gene and a ssDNA template containing an in-frame STOP codon (Supplementary Figure 1B and Supplementary Methods online). This approach should halt translation while concomitantly inducing nonsense-mediated mRNA decay (NMD) of the edited transcript. Introduction of the STOP codon was also designed to generate a new restriction site (*Ple*I) for subsequent evaluation of HDR editing by restriction fragment length polymorphism (RFLP) assay. Finally, the PAM sequence, a recognition motif required for Cas9 cleavage, was mutated in the template DNA to avoid repetitive cleavage of productively edited loci. The mutation of the PAM sequence was also designed to generate a second in-frame STOP codon to further ensure termination of translation (Supplementary Figure 1B and Supplementary Methods online). To further evaluate the need for PAM mutagenesis, ssDNA templates with or without PAM mutation were used at equal ratios in all described experiments.

HDR efficiency was subsequently evaluated in *ex vivo* cultured NSG embryos that were microinjected with gRNA#1, ssDNA and Cas9 at the zygote stage. A total of 13/65 (20%) embryos displayed productive repair by HDR as demonstrated by RFLP assay using *Ple*I (Figure 1a and Supplementary Figure 2). Moreover, T7 endonuclease assay, which reflects overall editing by detecting DNA mismatches, revealed that most embryos were edited in this context (Figure 1a, bottom panel). This suggests that, in addition to HDR, other repair mechanisms are likely at play. DNA-PK independent alternative end-joining (Alt-EJ) mechanisms that rely on short homologous sequences at the junctions have been described. This type of Alt-EJ often yields products with deletions (for example, #E7, Figure 1a). This is in line with a recent report demonstrating the occurrence of microhomology-mediated end joining in NSG mice.^10^

**Figure 1:**
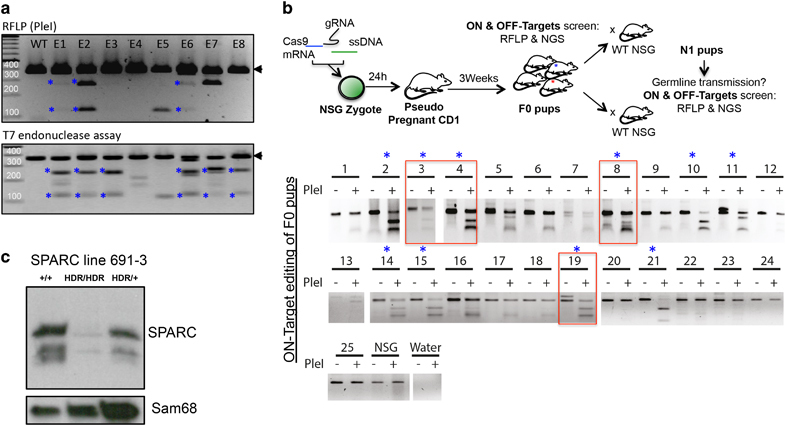
CRISPR/Cas9 allows for the fast and efficient generation of SPARC deficient NSG mice. (**a**) RFLP (*Ple*I digest) and T7 endonuclease analysis of day 4 NSG embryos microinjected with gRNA#1, Cas9 and ssDNA template. Asterisks mark productively edited embryos. (**b**) Schematic view of the workflow to generate NSG-edited line (top) and RFLP analysis of tail DNA from chimeric F0 mice (bottom). Asterisks mark HDR-edited mice. A subset of F0 mice (red squares) were backcrossed to WT NSG mice to obtain N1 progeny and evaluate germline transmission of the HDR+ allele. (**c**) Western blot on bone marrow cells shows complete loss of SPARC protein in SPARC^HDR/HDR^ animals, whereas a significant reduction is also observed in heterozygous SPARC^HDR/+^ mice. Sam68 is used as loading control.

Importantly, when microinjected NSG zygotes were implanted in pseudo-pregnant CD1 females, they gave rise to edited F0 progeny. RFLP analysis demonstrated that 40% of the mice (10/25) underwent productive HDR (Figure 1b). Importantly, this level of HDR editing in NSG zygotes is not restricted to the SPARC locus, as we observed comparable efficiencies when we targeted, using the same workflow, two other loci located on chromosome 5 (21.4% HDR+ F0 pups) and chromosome 8 (23.5% HDR+ F0 pups (Nevmerzhitskaya A and Medyouf H, manuscript in preparation). Of note, editing of the SPARC locus using the same workflow in C57BL/6 zygotes gave rise to a comparable frequency of 29% HDR-edited embryos (*n*=7/24, Supplementary Figure 3). Taken together, these data demonstrate that HDR is highly active in NSG mice and can be exploited for the rapid generation of knock-in and knockout mice.

To precisely define and quantify the editing, we amplified the predicted on- and off-target sites from all HDR+ F0 pups (Figure 1b, asterisk) and incorporated mouse-specific barcodes to be used as sample identifiers on the Illumina (San Diego, CA, USA) MiSeq platform (Supplementary Figure 4A and Supplementary Methods). F0 editing was highly efficient (mean edited variant allele frequency=75%) and mediated through either HDR (mean HDR+ variant allele frequency=35%) or Alt-EJ (mean variant allele frequency=30%) (Supplementary Figures 4B and C). Of note, edited sequences with and without PAM mismatch were observed at comparable frequencies in the F0 pups, thereby suggesting that PAM motif conservation in the ssDNA template does not decrease the frequency of productive HDR editing (Supplementary Figure 4B). Importantly, no editing was observed at predicted off-target sites in any of the analyzed mice. This was further confirmed by high coverage sequencing in the N1 progeny obtained from four of the F0 founders (#3, #4, #8 and #19; data not shown). We speculate that this lack of off-target editing might be contributed to by the use of a highly selective gRNA with limited predicted off-targets, as well as the inability of this strain to carry out error-prone NHEJ.

Notably, we used RFLP analysis to show that the HDR+ allele exhibited efficient germline transmission to the N1 progeny (Supplementary Figure 5A) in all four F0 founders tested (line 691, F0 founders #3, #4, #8, #19). To establish a SPARC line, we intercrossed the progeny of 691#3 carrying the HDR allele after HDR-mediated editing was further confirmed by standard Sanger sequencing (data not shown). Heterozygous mating demonstrated Mendelian transmission (Supplementary Figure 5B). Molecular analysis showed that both SPARC RNA and protein were absent from homozygous SPARC^HDR/HDR^ (referred to as SPARC-deficient) mice, indicating that the mRNA produced from the HDR-edited allele is likely degraded through NMD *in vivo*, a well known translation-coupled mechanism that eliminates mRNAs containing premature translation-termination codons (Figure 1c and Supplementary Figure 5C). Finally, similar to their C57BL/6 counterpart, NSG SPARC-deficient mice appear normal until ∼4–6 months of age, when they develop severe cataract formation (Supplementary Figure 6).^11^

Because mouse and human SPARC have a 92% sequence identity at the protein level, we used our NSG SPARC-deficient model to interrogate *in vivo* the specific effect of niche-derived SPARC in human leukemia. Initially, we focused on acute myeloid leukemia (AML), where seemingly conflicting results have been reported. Indeed, high SPARC expression has been shown to associate with adverse outcome in cytogenetically normal AMLs,^6^ whereas SPARC appears to rather reduce the growth of low SPARC expressing MLL-rearranged (MLL^R^) AML blasts *ex vivo*.^7^ However, thus far, no study has investigated the role of niche-derived SPARC in the pathogenesis of human AML *in vivo*. When compared with wild-type mice, NSG SPARC^HDR/HDR^ animals injected with THP-1, a SPARC^low^ MLL-AF9^+^ line,^7^ exhibited a dramatically increased leukemic burden both at an early time point post transplant (Figure 2b) and in terminally ill animals (Supplementary Figure 7). Conversely, xenotransplantation of a cytogenetically normal primary AML case in our model rather showed a tendency toward lower burden in SPARC-deficient animals (Figure 2b).

**Figure 2:**
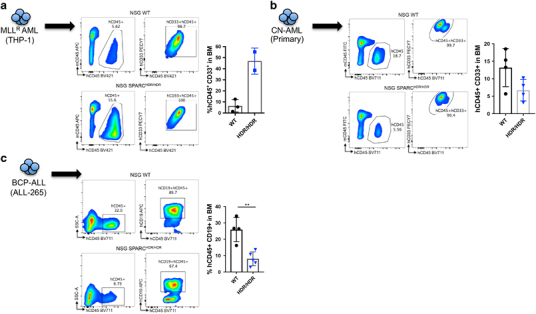
NSG SPARC mice reveal opposing effects of niche-derived SPARC in human leukemia. Percentage and immunophenotyping of hCD45^+^ cells in the bone marrow of WT and SPARC^HDR/HDR^ mice engrafted with 10^6^ MLL-AF9-expressing THP-1 cells (WT NSG *n*=3; SPARC KO *n*=2) analyzed 5 weeks post transplant (**a**), 10^6^ cells from a cytogenetically normal (CN) primary AML case (WT NSG *n*=4; SPARC KO *n*=4) analyzed 11 weeks post transplant (**b**) or 10^5^ cells from a BCP-ALL relapsed case (ALL-265; WT NSG *n*=4; SPARC KO *n*=5) analyzed 5 weeks post transplant (**c**). The data show a significantly increased leukemic burden in SPARC^HDR/HDR^ mice receiving THP-1 cells (**a**). In stark contrast, leukemic burden is dramatically decreased in SPARC^HDR/HDR^ mice receiving the primary CN-AML case or the relapsed BCP-ALL case (ALL-265) (**b**, **c**). Of note, additional THP-1 and BCP-ALL cases (ALL-199) are depicted in supplementary Figures 7 and 8. Each dot represents an independent mouse. Unpaired Student’s *t-*test, **P<0.01.

Similarly, divergent roles for SPARC have been proposed in lymphoid malignancies, where absence of stromal SPARC predicts poor prognosis in diffuse large B-cell lymphoma,^12^ whereas its leukemia-specific upregulation is a recurrent event in relapsed pediatric B-cell acute lymphoblastic leukemia (B-ALL).^13^ We therefore used PDX to evaluate the impact of exogenous SPARC in relapsed pediatric B-cell precursor ALL cases (BCP-ALL; ALL-265 and ALL-199).^14^ In stark contrast to the THP-1 model, SPARC deficiency dramatically decreased the BCP-ALL burden in mice (Figure 2c and Supplementary Figure 8), thereby suggesting that tumor microenvironment-derived SPARC can elicit downstream signals that effectively promote the growth of an aggressive form of BCP-ALL.

Taken together, these data clearly highlight *in vivo* that niche-derived SPARC modulates leukemic cell behavior. Most importantly, this effect appears to be highly specific to the cellular context.

This work describes a new strategy to generate genetically modified NSG mice that are of great value to interrogate the functional relevance of key niche factors in PDX models *in vivo*. In addition to this technical aspect, the approach was also successfully used to demonstrate, for the first time, the divergent functions of niche-derived SPARC in human leukemia in an *in vivo* setting. Future studies will explore the disease-specific programs that mediate SPARC downstream effects *in vivo.* Identifying such programs might pave the way to the design of new therapeutic strategies, in particular for patients in urgent need for alternative therapies.

## Supplementary information

Supplementary Material (DOCX 27502 kb)
