# Supplementary material for: CRISPR/Cas9-edited NSG mice as PDX models of human leukemia to address the role of niche-derived SPARC
Source: Leukemia. 2017 Dec 6;32(4):1048–51. doi: 10.1038/leu.2017.346 (PMC7703605; doi:10.1038/leu.2017.346)
Supplement: Supplementary file 1 — Supplementary Material (DOCX 27502 kb) [file 41375_2018_BFleu2017346_MOESM1_ESM.docx]

**CRISPR/Cas9 edited *NSG* mice as PDX models of human leukemia to address the role of niche-derived SPARC**

Tirado-Gonzalez I^1,*^, Czlonka E^1,*^, Nevmerzhitskaya A^1,*^, Soetopo D^1^, Bergonzani E^1^, Mahmoud A^1^, Contreras A^1^, Jeremias I^2,3^, Platzbecker U^4,5^ , Bourquin JP^6^, Kloz U^6^, Van der Hoeven F^7^, and Medyouf H^1,8^.

**Supplemental data ­**

**Supplementary material and methods**

**Human primary samples and animal experiments**

PDX BCP-ALLs (ALL-265 and ALL-199) have been described previously^1^. Clinical information is provided in supplemental table 3. All patient samples were obtained after informed consent. All xenotransplant experiments were carried out in mice without prior conditioning and approved by the Regierungspräsidium Darmstadt under animal protocol numbers F123/1008 and F123/1034. Experimental animals were bred at the animal facility of the Georg-Speyer-Haus in IVCs under specific pathogen free conditions. 8-12 weeks old mice were used in functional assays. Experimental groups were sex and age matched. Mice were analyzed at the time point indicated in figure legends. No blinding was performed.

**Statistical analysis**

Statistics were performed with Prism statistical software (GraphPad). Unpaired Student’s t-test was used to compare two groups in all experiments. Shapiro-Wilk test was used to test for normal distribution. Significant outliers were identified and removed based on the Grubb’s test with significance level alpha set to 0.05. Details for each analysis are in figure legends.

**Flow cytometry analysis of xenografted mice**

Assessment of engraftment was carried out using bone marrow cells isolated from femur and tibiae of injected mice, after red blood cell lysis using ACK lysis buffer (Lonza). Cells were stained on ice for 30 minutes, at 4°C with the following antibodies using optimized dilutions: hCD45-PE (BD Biosciences, Clone HI30; 1:100) or hCD45 BV421 (BD Biosciences, Clone HI30, 1:100) or hCD45 BV711 (BD-Biosciences, Clone HI30, 1:300); hCD33-PECY7 (BD Biosciences, Clone WM-53; 1:100); mCD45.1 FITC (eBioscience, clone A20, 1:300) or mCD45 APC (BD Bioscience, clone 30-F11, 1:100), hCD19-APC (eBioscience, clone HIB19, 1:100); hCD10-APCVio770 (MiltenyiBiotec, Clone 97C5, 1:100). All stains contained both human Fc Block (MiltenyiBiotec; 1:20) and mouse Fc Block (CD16/32 purified, BD Biosciences; 1:500). Data were acquired on a BD Fortessa (BD biosciences) and analyzed using FlowJo software (Tristar). Dead mononuclear cells were gated out using Propidium Iodide (Invitrogen) after doublets exclusion or Hoechst staining.

**RNA extraction and qRT-PCR.**

Total RNA was isolated from bone marrow mononuclear cells using the Qiagen RNAeasy kit following the manufacturer’s instructions (Qiagen). cDNA was generated using the High Capacity cDNA RT Kit (Applied Biosystems) according to the manufacturer’s instructions. Quantitative RT-PCR was performed with the ABI Power SYBR Green Master Mix (Life Technologies). PCR reactions were performed on a Viia7 (Life Technologies) with the following primers: *mSparc fw:* AATTTGAGGACGGTGCAGA; *mSparc-rv: CTCACACACCTTGCCATGTT;* m*Sdha* fw: AAGTTGAGATTTGCCGATGG; *mSdha* rv: TGGTTCTGCATCGA CTTCTG; m*Oaz1*-fw: TTTCAGCTAGCATCCTGTACTCC; m*Oaz1*-rv: GACCCTGGTCTTGTCGTTAGA. *Sdha* and *Oaz1* were used as endogenous references.

**sgRNA design and cloning**

MIT CRISPR software (<http://crispr.mit.edu>) was used to design 2 gRNAs that directed Cas9 to a translated sequence in exon 4 (gRNA1# CGGTGCAGAGGAAACGGTCG) or a partially translated region of exon 2 (gRNA2: TCTCCTTTGCCTGGCCGGGA) of the *Sparc* gene. Single guides were independently cloned into the previously described pX330-U6-Chimeric_BB-CBh-hSpCas9 (gift from Feng Zhang, Addgene plasmid # 42230) as described previously ^2^.

**Cell lines**

MS5 cells were grown in a-MEM medium supplemented with 10% FBS (Gibco), penicillin-streptomycin (Invitrogen), 5% glutamine (Invitrogen) and 5% sodium pyruvate (Invitrogen). The MLL-AF9 expressing acute myeloid leukemia cell line, THP-1, were a kind gift from Dr. Stefan Stein and were maintained in RPMI-1640 supplemented with 1% P/S, 1% HEPES, 1% Glutamine in the presence of 50uM 2-mercaptoethanol and 10% FBS. The cells were tested for mycoplasma contamination and authenticated using short tandem repeats (SRF) profiling by the German Biological Resource Centre DSMZ.

**Ex-vivo evaluation of sgRNAs efficiency by surveyor assay**

MS5 cells were transfected with either gRNA-expressing pX330 (gRNA1 or gRNA2) or empty vector, using a Calcium Phosphate Transfection Kit (Invitrogen). Three days post transfection genomic DNA was extracted using the DNeasy Blood and Tissue Kit (Qiagen) according to the manufacturer instructions. Unique primers were designed to span the expected DSB sites and amplify a region of about 400bp. For gRNA2 primers were as follows: mSPARCex2-fw 5’-AGGAGGTACAAAGGGCGTCT-3’; mSPARCex2-rv 5’-CGCTGGGATTGTAGGTTTGT-3’. For gRNA1 primers were as follows: mSPARCex4-fw 5’-AATTCCACCAAAACCAGCAC-3’; mSPARCex4-rv 5’-CCACAGCTACCAGAGCTTCC-3’. All PCRs were carried out using the Phusion High Fidelity DNA Polymerase (M0530S, New England BioLabs) and products were purified using the QIAquick PCR Purification Kit (Qiagen). Editing was evaluated using the Surveyor® Mutation Detection Kit for Standard Gel Electrophoresis according to the manufacturer’s instructions (IDT technologies). Briefly, PCR products corresponding to gRNA1 or gRNA2 edited cells were mixed with the reference PCR product obtained from pX330 control transfected cells, in equal amounts (100ng each). The mixture was subjected to a hybridization reaction as follow (10min, 95°C; ramp down to 85°C at -2°C /s; ramp down to 25°C at -0.3°C /s; hold at 4°C). DNA heteroduplexes were then digested by CEL endonuclease according to the manufacturer’s instructions and analyzed on a 2% agarose gel.

**Generation of SPARC-deficient NSG mice**

SPARC knock-out mice were directly generated using NSG mice obtained from The Jackson Laboratories (Jackson laboratories; line: 005557). Genome editing using CRISPR/Cas9 was approved by the Regierungspräsidium Karlsruhe under animal protocol number G50/15.

pX330-U6-gRNA1_BB-CBh-hSpCas9 was used as a template to generate gRNA1 using the MEGAshortscript T7 transcription kit following the manufacturer’s instructions. Cas9 mRNA was purchased from Tebu-Bio. For cytoplasmic microinjection of fertilized NSG zygotes, gRNA1 was used at a final concentration of 50ng/μl and individually mixed with Cas9 mRNA at 100ng/μl, with or without ssDNA template at a final concentration of 50ng/μl in nuclease free water. ssDNA templates with and without PAM mismatch were used at equal concentration. Both templates were designed to introduce an in-frame STOP codon (***TGA****) and a PleI restriction site (GAGTCCGAGT) with (ssDNA1) or without (ssDNA2) PAM mismatch (AG**T^#^**). ssDNA were obtained from IDT as 4nmole Ultramer® DNA Oligos.

ssDNA1: GTGGGTGCCAACCCAGTCCAGGTGGAAATGGGAGAATTTGAGGA*CGGTGCAGAGGAAACG****TGA****GTCGAG**T^#^**AGGTGGTGGCTGACAGTAAGTCTCTTCCATGTCACTTGGCGTATCTGGATCACCCCCAGC

ssDNA2: GTGGGTGCCAACCCAGTCCAGGTGGAAATGGGAGAATTTGAGGA*CGGTGCAGAGGAAACG****TGA****GTCGAGGAGGTGGTGGCTGACAGTAAGTCTCTTCCATGTCACTTGGCGTATCTGGATCACCCCCAGC

NSG zygotes were obtained from 8-10 week old superovulated NSG female donor mice mated with NSG stud males and fertilized embryos (zygotes) were collected from oviducts. Ca. 10-20 pl of reagents were injected in the cytoplasm of zygotes. The same day, injected embryos were implanted into 0,5 dpc. pseudo-pregnant CD1 foster mothers. For embryos analysis, injected zygotes were cultured in KSOM media for 4 days at 5% CO2, 37°C, prior to genomic DNA analysis.

**Embryos and mice screening by RFLP and T7 endonuclease assay**

Day 4 NSG embryos were individually lysed in a total volume of 9.5μl of DirectPCR® Lysis-Reagent Cell (peqlab) plus PCR grade proteinase K at 125μg/ml (Roche) according to the manufacturer’s instructions. The first PCR was performed with 5μl input material (whole embryo lysis) using primers that span the expected DSB site and amplify a region of 400bp *mSparc*-ex4-fw 5’-AATTCCACCAAAACCAGCAC-3’; *mSparc*-ex4-rv 5’-CCACAGCTACCAGAGC TTCC-3’. Because of the limited starting material, a second nested PCR was carried out using the flowing primers: *mSparc*-ex4Nest-fw CCACCAAAACCAGC ACCT; *mSparc*-ex4Nest-rv GCATGCCCCTGACTAGAAAC and yielded a 370bp product. For mice, genomic DNA was directly isolated from tail biopsies using the DNeasy Blood and Tissue Kit (Qiagen) following the manufacturer’s instructions and *mSparc*-ex4-fw and *mSparc*-ex4-rv used to amplify a 400bp product spanning the DSB site. PCR1 with *mSparc*-ex4-fw and *mSparc*-ex4-rv was carried out as follows: 98°C 1 min [98°C 30s; 66°C 30s; 72°C 15s] x 35; 72°C 7min; 4°C hold step. For embryos, 1μl of PCR1 was used as template for PCR2. PCR2 with *mSparc*-ex4Nest-fw and *mSparc*-ex4Nest-rv was carried out as follow: 98°C 1 min [98°C 15s; 63°C 30s; 72°C 20s] x 24; 72°C 7min; 4°C hold step.

**Restriction Fragment Length Polymorphism** assay was carried out using PleI enzyme (New England Biolabs) and 500ng of purified PCR product for 1h at 37°C as *per* manufacturer’s instructions. After heat inactivation for 20min at 65°C, the products were resolved on a 3% agarose gel. **For the T7 endonuclease 1 assay**, PCR products obtained from WT mice (or embryos) were mixed with the ones obtained from edited mice (or embryos) in equal amounts (100ng each). 200ng of purified PCR product were mixed with 2ul NEBuffer 2 and dH2O to a total of 19.5μl in a PCR tube followed by a hybridization reaction (5min, 95°C; ramp down to 85°C at -2°C /s; ramp down to 25°C at -0.1°C /s; hold at 4°C). 0.5μl (5U) T7 endonuclease I (M0302, New England BioLabs) was added and the mixture incubate at 37C for 2h. After T7 endo I inactivation with EDTA, the products were analyzed on a 3% agarose gel (16500-500, Sigma-Aldrich). In both assays, amplicons edited with HDR yielded two fragments of 260bp and 140bp.

**Sanger sequencing from tail DNA**

PCR products using primers flanking the targeted site (*mSparc*-ex4-fw and *mSparc*-ex4-rv as above) were directly subcloned into CloneJET PCR Cloning Kit (Life technologies) according to the manufacturer’s instructions. Plasmids were transformed into chemically competent One Shot ^TM^ Stbl3^TM^ *E.coli.* Single bacterial colonies were then grown in LB media plus Ampicillin. Plasmid DNA isolated using the QIAprep spin Miniprep Kit (Qiagen) was then subjected to standard Sanger sequencing (GATC Biotech, Germany).

**Library preparation for next generation sequencing of On & Off-Target sites**

Genomic DNA from tail biopsies was used to amplify specific gDNA regions using primers that bind sequences flanking the predicted On and Off-target DSB sites. The PCR1 cycling conditions were as follows (MiSeqPCR1: 98°C 1 min [98°C 10 s, 65°C 30 s, 72°C 30 s] x35, 72°C 5 min, 4°C hold step). The primers used in MiSeqPCR1 were composed of two parts: the target specific sequence and common adapter sequences: MiSeqF 5’–TCGTCGGCAGCGTCAGATGTGTATAAGAGACAG-Target_specific_Primer–3’ & MiSeqR 5’–GTCTCGTGGGCTCGGAGATGTGTATAAGAGACAG-Target_Specific_Primer–3’ (List of all MiSeq primers in supplementary table 2). The MiseqF adapter was linked to a target specific primer that is about 75bp (50-100bp) from the predicted CRISPR site. In order to maximize sample sequencing on the Illumina MiSeq platform, PCR amplicons from MiSeqPCR1 were individually purified using 1.8x reaction volume of AMPure XP beads (Beckman Coulter). Adapter sequences incorporated in MiSeqPCR1 were then used for IndexPCR2 using Illumina’s Nextera XT Indexing Primers (Illumina) to incorporate sample specific identifiers compatible with the Illumina MiSeq platform. IndexPCR2 was carried out using an equimolar amount of all On and Off target amplicons obtained in PCR1. The PCR2 cycling conditions were as follows (IndexPCR2: 98°C 1 min [98°C 10 s, 65°C 30 s, 72°C 30 s] x12, 72°C 5 min, 4°C hold step). IndexPCR2 products were purified using 1.8x reaction volume of AMPure XP beads (Beckman Coulter), quantified using Life Technologies Qubit Fluorometer dsDNA High-Sensitivity kit and amplicon size distribution was ascertained using the Agilent Tape-station using the D1000 protocol. All PCRs were carried out using NEBNext® High-Fidelity 2X PCR Master Mix (New England Biolabs).

**Illumina MiSeq sequencing and next generation sequencing (NGS) data analysis**

Each dual indexed library was diluted to 12pM for sequencing on a MiSeq instrument using the 300-cycle kit following the manufacturer’s instructions (Illumina). After generating the raw data for the ON and OFF target sites, we used Trimmomatic (Version 0.36) for trimming the indexes from the sequencing reads. Then we used [Burrows-Wheeler Aligner](https://sourceforge.net/projects/bio-bwa/) (Version 0.7.12-r1039) for mapping reads to the mouse reference genome (Mus_Musculus GRCm38). We used Picard (Version 2.2.1) for converting SAM to BAM files, read group and indexing. Finally, we used Genome Analysis ToolKit (GATK) (Version 3.5) for local realignment and calling variants with haplotypeCaller, and used Integrative Genomics Viewer (IGV) for visualizing the ON and OFF target sites.

**Western blot analysis**

Western blot analyses were performed as follows. Briefly, total protein extracts were prepared from bone marrow mononuclear cells using RIPA buffer supplemented with phosphatase inhibitor (1:100, P5726, Sigma-Aldrich) and protease inhibitor (1:100, P8340, Sigma-Aldrich) cocktails. A goat polyclonal antibody was used to detect SPARC (1:1000, AF942, R&D Bioscience). A rabbit polyclonal antibody against SAM68 was used as loading control (1:1000, sc-333, Santa Cruz Biotechnologies). Secondary Horseradish peroxidase-conjugated anti-goat (sc-2020, Santa Cruz Biotechnologies; 1:2000) and anti-rabbit (NA934V, GE-Amersham Biosciences, 1:5000) were used.

**Allele specific genotyping protocol**

The new NSG SPARC^HDR/HDR^ line (691-3) was genotyped using the following primers: NSG_SPARCwt_Fw2: ACGGTGCAGAGGAAACG**GTC**; NSG_SPARCwt_RV: CAGACTTCCCTGGCTTGAGG amplifying a 200bp product from the wild type allele. NSG_SPARCKO_Fw: ACGGTGCAGAGGAAACG**TGA** and NSG_ SPARCKO_Rv: CATGGCTTTGGACACGGATG amplifying a 490bp product from the HDR^+^ edited allele (knock out). The PCR was carried using DreamTaq DNA polymerase (ThermoFisher Scientific) and the following program (95°C 5 min [95°C 15 s, 60°C 30 s, 72°C 1 mn] x35, 72°C 5 min, 4°C hold step).

**Supplementary references**

1. Ebinger S, Ozdemir EZ, Ziegenhain C, Tiedt S, Castro Alves C, Grunert M*, et al.* Characterization of Rare, Dormant, and Therapy-Resistant Cells in Acute Lymphoblastic Leukemia. *Cancer cell* 2016 Dec 12; **30**(6)**:** 849-862.

2. Cong L, Ran FA, Cox D, Lin S, Barretto R, Habib N*, et al.* Multiplex genome engineering using CRISPR/Cas systems. *Science* 2013 Feb 15; **339**(6121)**:** 819-823.

**Supplementary tables and figures**

**Supplemental Table 1 List of predicted Off-targets**

**Supplemental table S1:** List of all exonic off-targets predicted by the MIT CRISPR software for the two gRNAs targeting the *Sparc* gene used in this study. *In our study, nine out of the ten HDR^+^F0 pups (marked with an asterisk in Figure 1B) were sequenced and analyzed for off-target editing. All sequenced samples failed to reveal any off-target editing. This finding was further confirmed by sequencing the HDR^+^ progenies obtained from F0 mouse number #3 (n=5 N1 and n=7 N1F1 progenies) and mouse #19 (n=4 N1 progenies) with similar results*.

**Supplemental table S2 List of all MiSeq primers used for NGS.**

**Supplemental table S3 Clinical data of human samples**

**Figure S1**

**
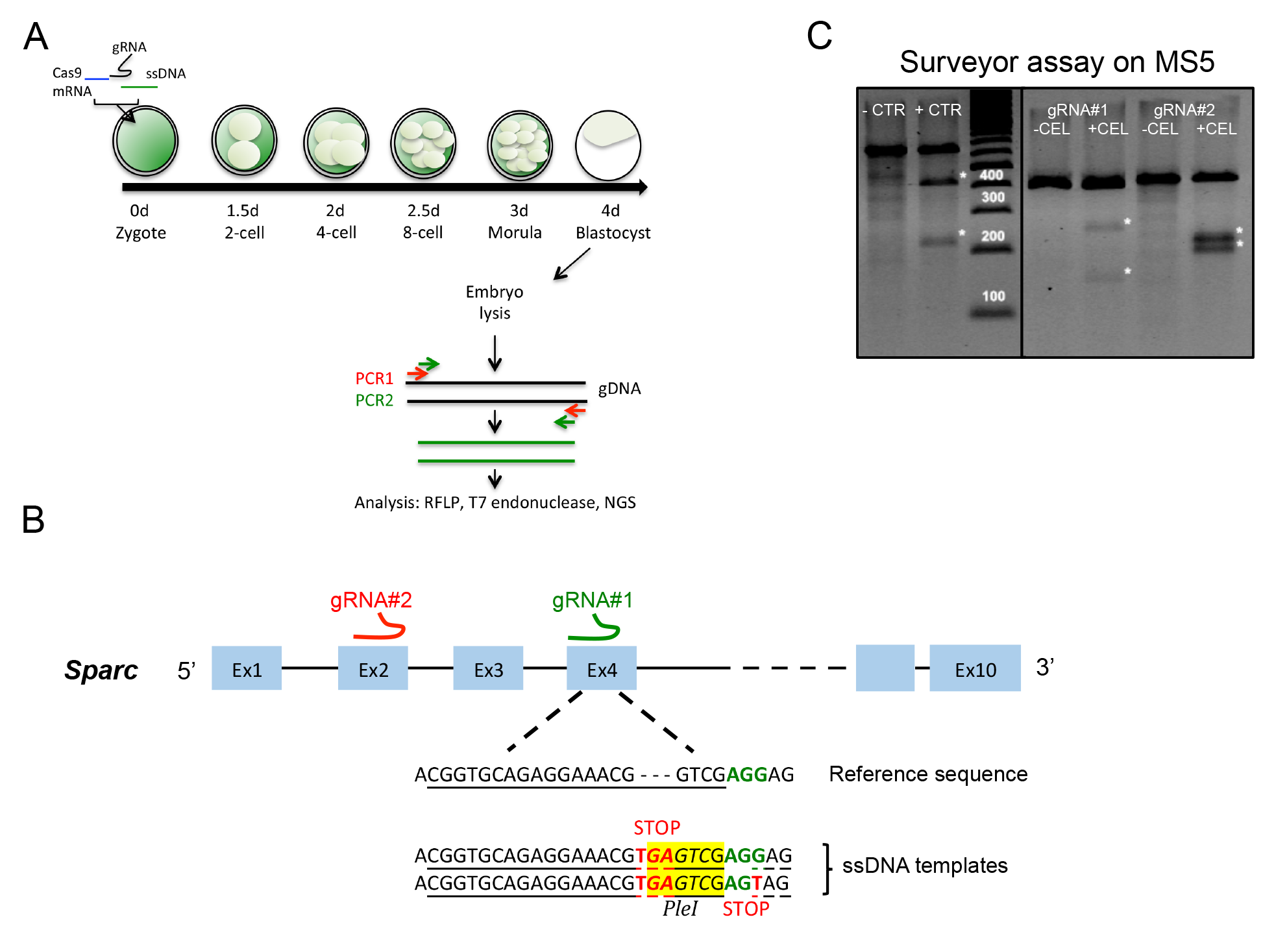
Supplemental figure 1(a)** Schematic illustration of the cytoplasmic microinjection in NSG zygotes and following analysis of NSG embryos at day 4 post ex-vivo culture. **(b)** Schematic view of the *Sparc* gene highlighting the translated exons targeted by the designed gRNAs. The region flanking the site targeted by gRNA1 within exon4 is depicted along with the mutated sequences of the ssDNA used as template for the HDR (the complete ssDNA sequence is provided in supplemtary material). The sequence targeted by gRNA1 is underlined both in the *Sparc* reference sequence and the ssDNA templates with and without PAM (AGG) mismatch. Insertion of the STOP codon also generated a PleI site. **(c)** Surveyor assay on DNA obtained from MS5 cells transfected with pX330 expressing Cas9 and either gRNA#1 or gRNA#2 directed to the *Sparc* gene. Productively edited alleles are cleaved and generate shorter bands indicated by an asterisk. Data indicate that both guides induce productive editing but also suggest gRNA#2 to be more effective than gRNA#1. Left panel are endogenous controls from the surveyor assay.

**Figure S2**

**
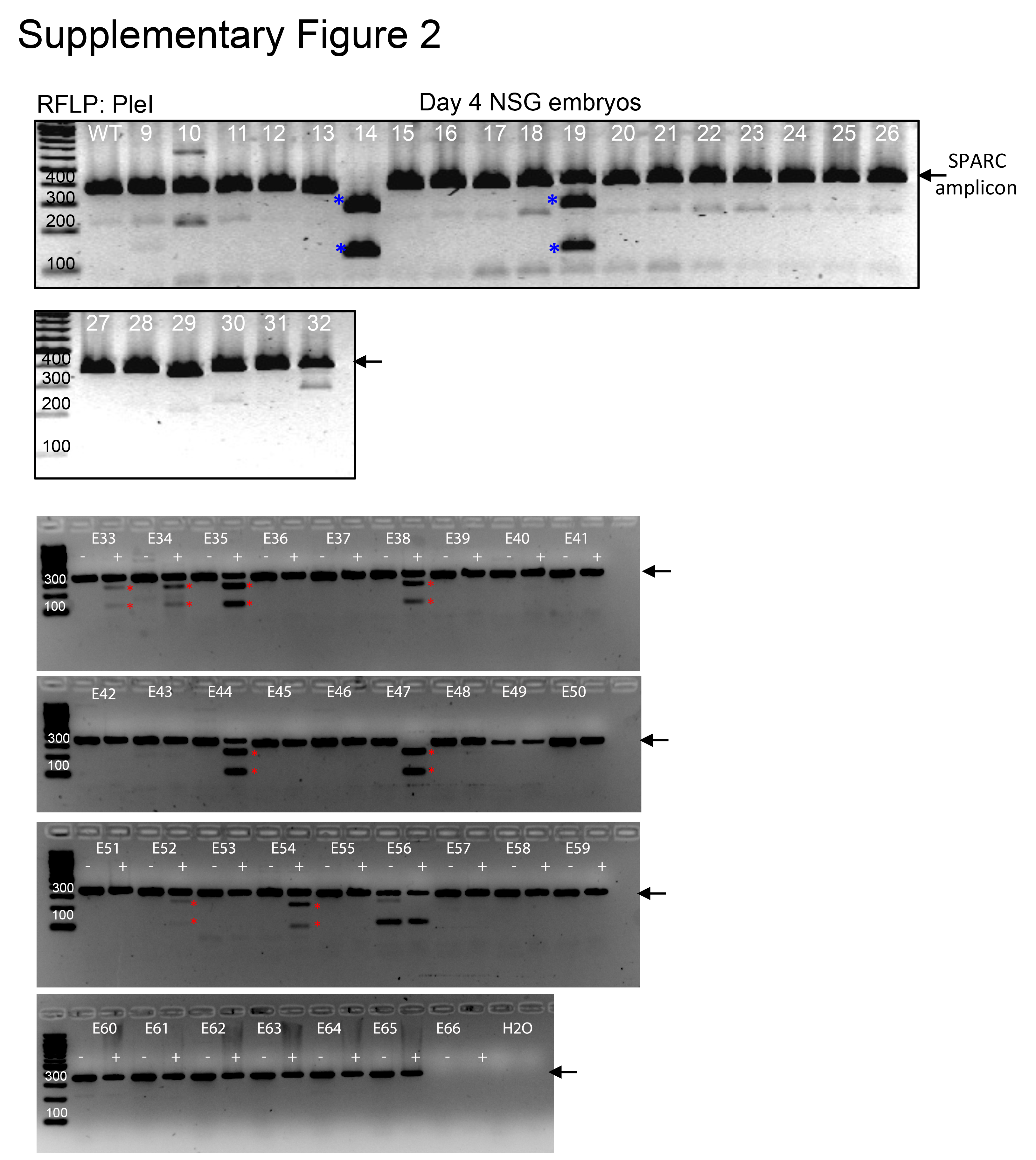
**

**Supplemental figure S2** Results of RFLP analysis of NSG embryos 4 days post cytoplasmic injection of NSG zygotes with Cas9 mRNA/gRNA#1 and ssDNA template. HDR edited embryos are marked with an asterisk. Of note, E14 and E47, show complete amplicon cleavage by PleI, which indicates that productive HDR editing occurred at the zygote stage (1 cell) on both alleles, in these embryos. A reference DNA ladder is loaded in the first lane.

**Figure S3**

**
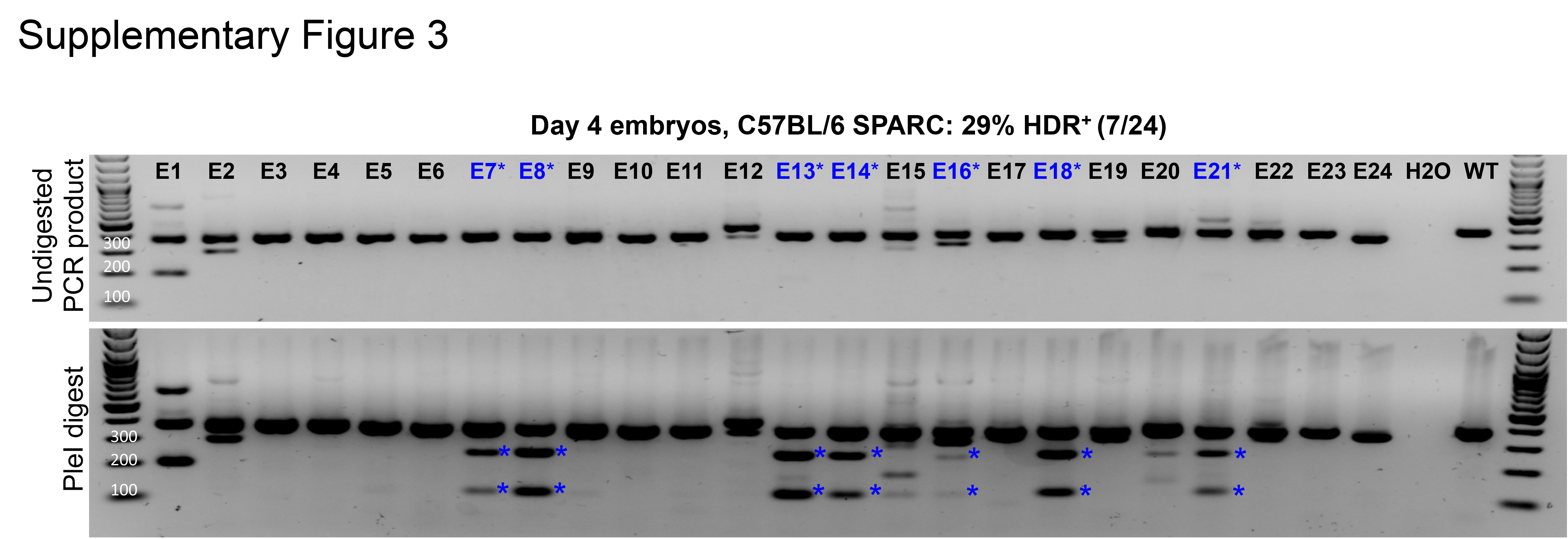
 Supplemental figure S3** Results of RFLP analysis of embryos 4 days post cytoplasmic injection of C57BL/6 zygotes with Cas9 mRNA/gRNA#1 and ssDNA template. HDR edited embryos are marked with an asterisk. DNA ladder are loaded in first and last lanes.

**Figure S4**

**
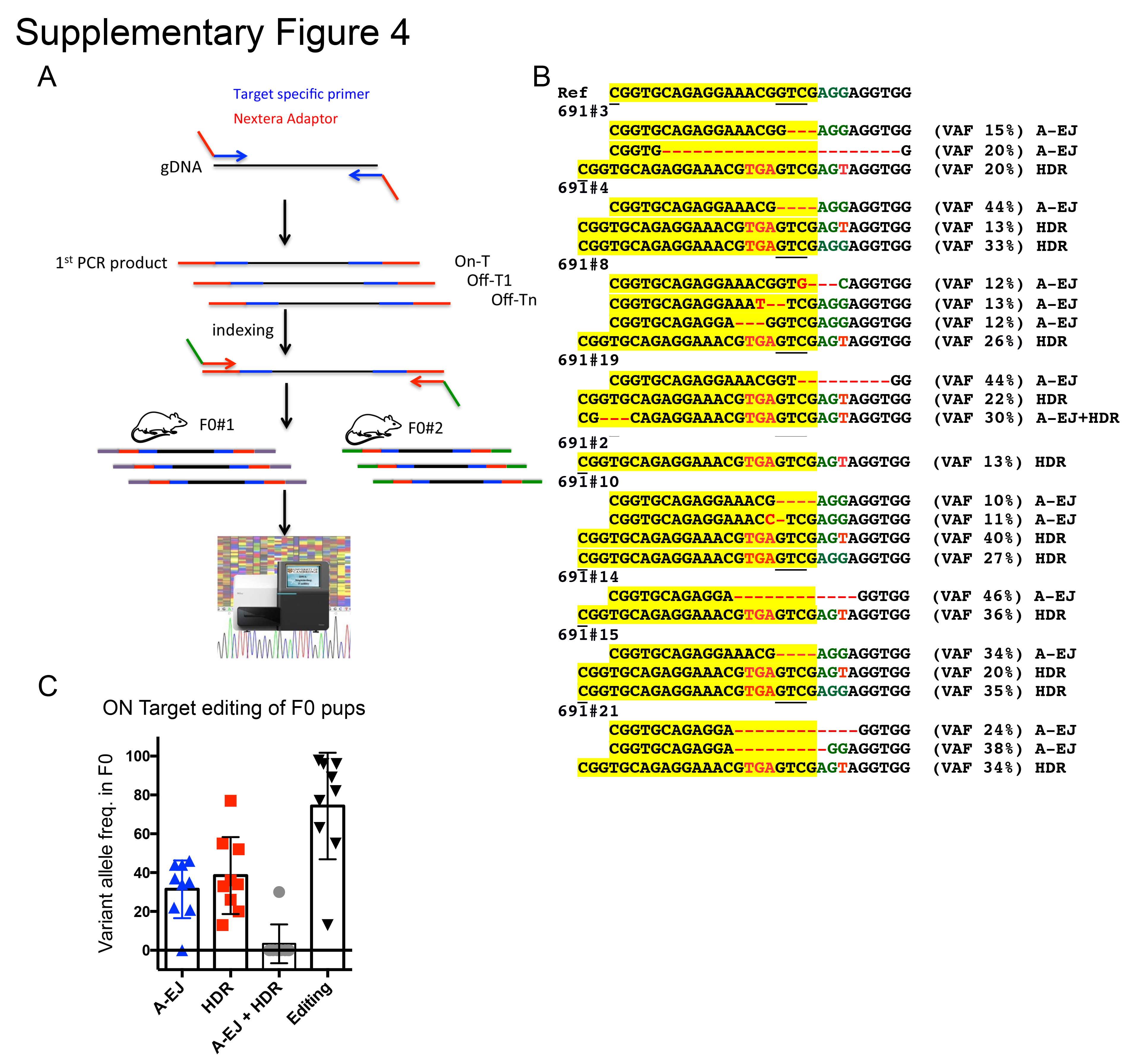
 Supplemental figure 4 : (a)** Schematic workflow and experimental design used in the next generation platform. gDNA is subjected to a first PCR using primers flanking the sgRNA binding sites (both On and predicted off targets). This PCR also introduces adapters that are used for the second PCR with the Nextera XT indexing primers for tracking of sample specific amplicons and next generation sequencing using MiSeq. **(b)** NGS data from nine out of ten SPARC F0 mice with productive HDR editing as demonstrated by RFLP analysis (Figure 1B). All alleles with a variant allele frequency >5% are indicated. Most F0 animals appear to carry at least one allele edited by productive HDR but also display variants that are likely generated by A-EJ repair mechanisms. **(c)** Summary of editing frequencies observed in n=9 HDR+ F0 mice using the NGS platform. Each dot represents an independent mouse. A-EJ and HDR mediated editing equally contribute to the tremendously high level of over-all editing observed in these chimeric F0 mice.

**Figure S5**

**
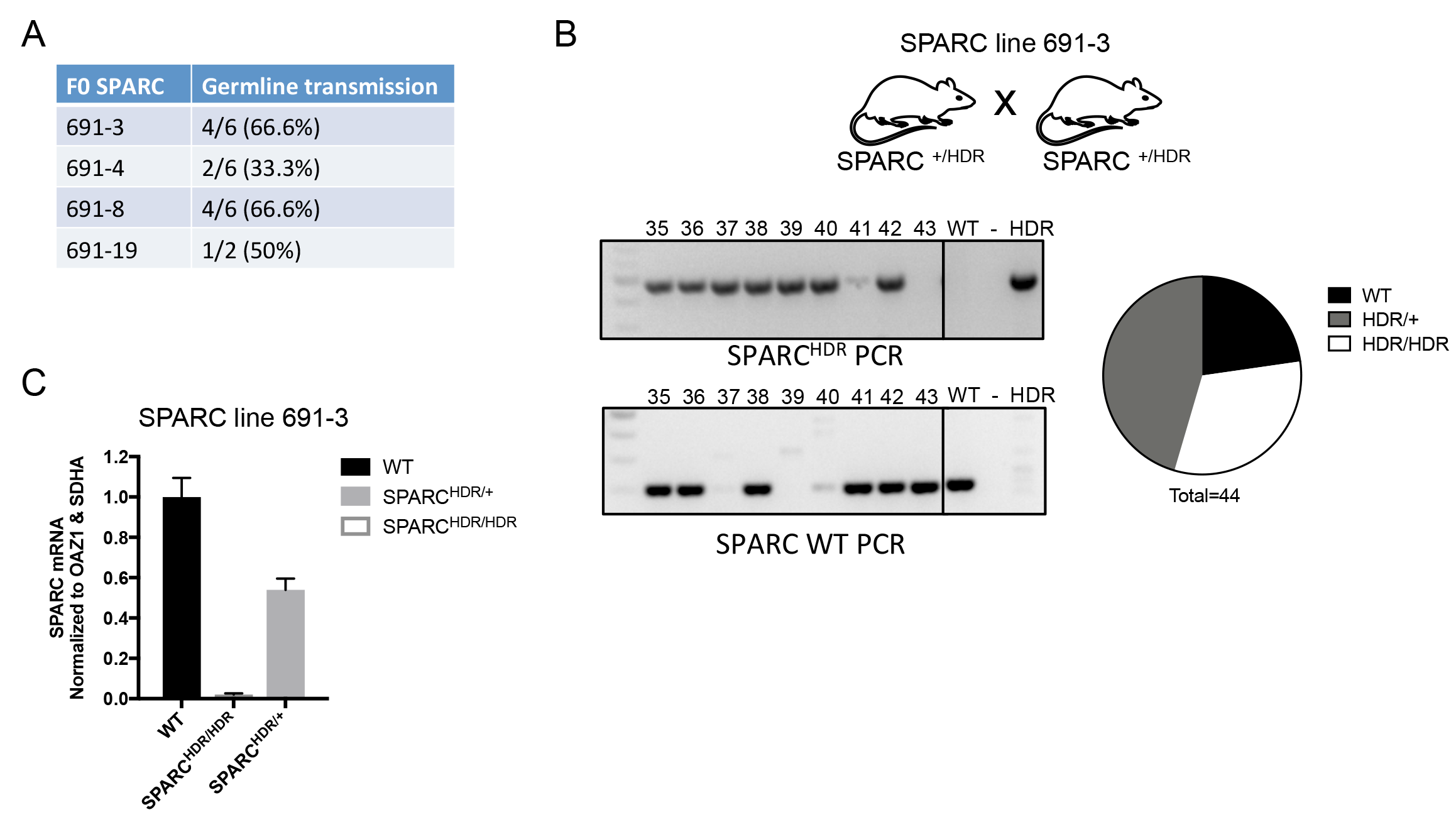
**

**Supplemental figure 5** Four HDR^+^ F0 NSG SPARC mice (691-3, -4, -8, -19) were backcrossed with NSG mice and germline transmission of the HDR^+^ allele was evaluated in the N1 progeny by RFLP assay and summarized in **(a)**. **(b)** Analysis of n=44 pups from heterozygous N1 mating (line 691-3), using an allele-specific genotyping protocol (Supplementary methods), demonstrate Mendelian inheritance of the HDR allele. **(c)** qRT-PCR demonstrates a complete loss of SPARC mRNA in bone marrow mononuclear cells from NSG SPARC^HDR/HDR^  animals. SPARC expression is also reduced by 50% in heterozygous animals. The data shown represent the mean and SD from n=3 independent replicates.

**Figure S6**

**
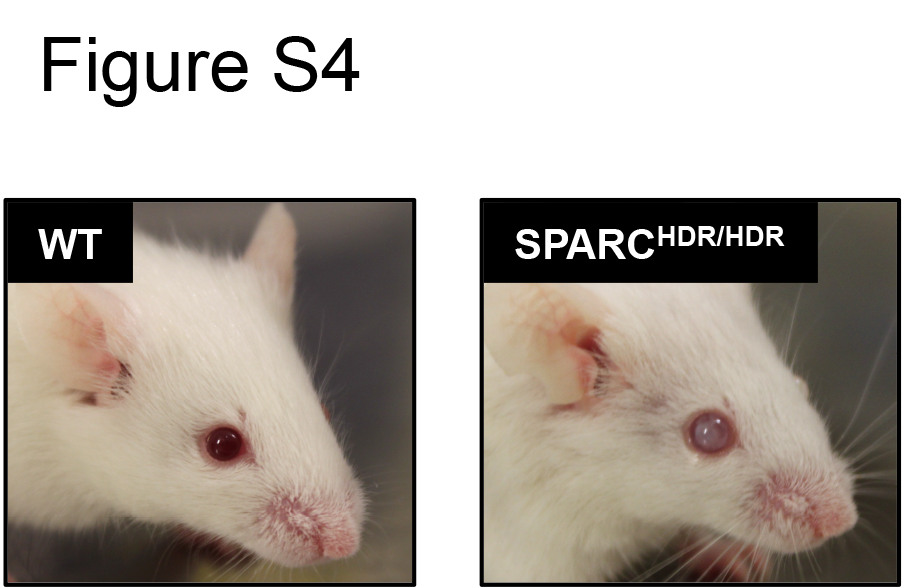
**

**Supplemental figure 6** Gross appearance of lens cataract in NSG SPARC deficient mice at 6 months of age.

**Figure S7**

**
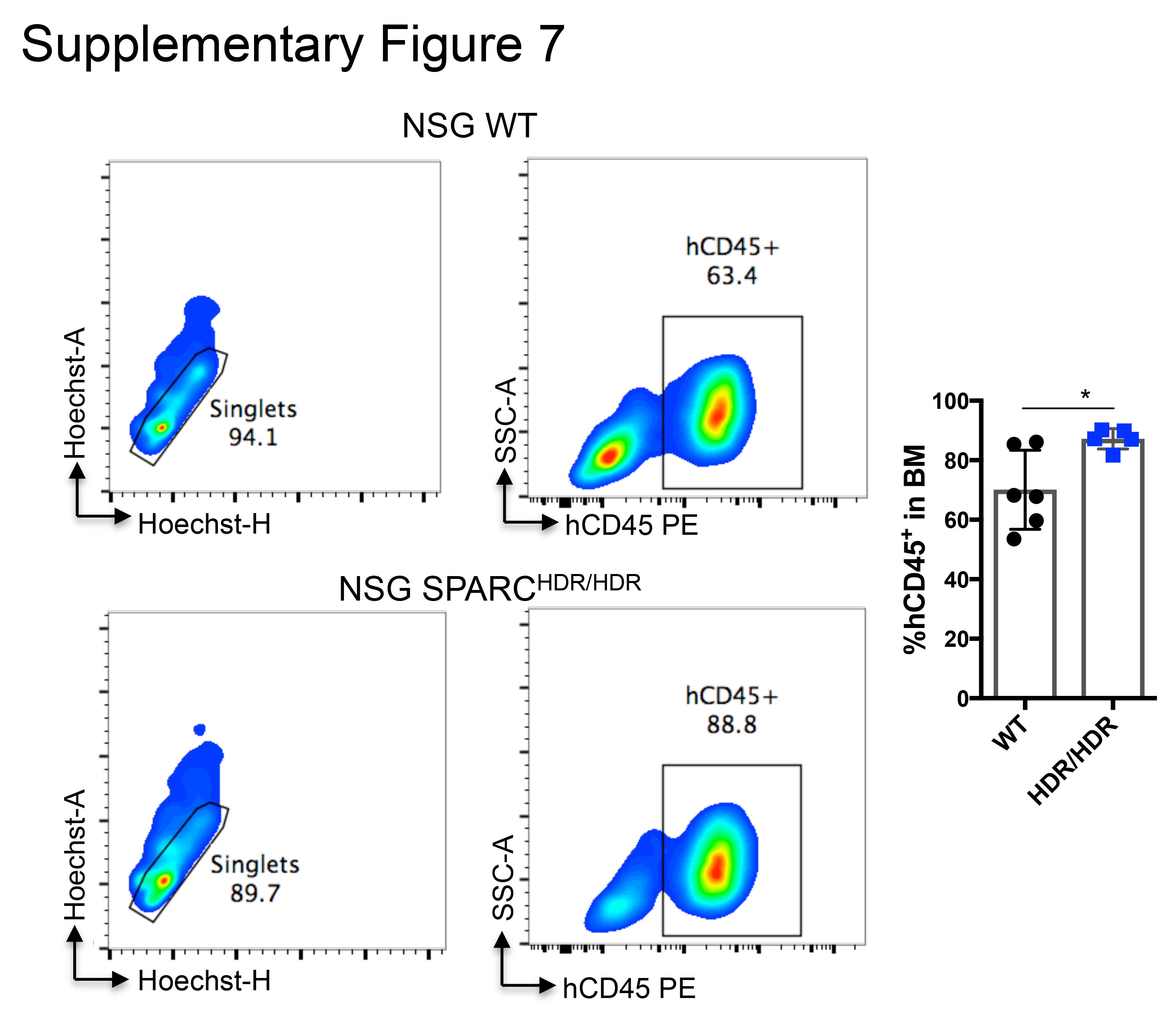
**

**Supplemental figure 7** Representative gating schemes and percentage of human CD45^+^~~CD33+~~ cells in the bone marrow of terminally–ill WT (n=6) and SPARC^HDR/HDR^ (n=5) NSG mice engrafted with 10^6^ MLL-AF9 expressing THP-1 cells. Mice were analyzed 8 weeks post transplant. The data show a significantly increased leukemic burden in SPARC^HDR/HDR^ mice. Each dot represents an independent mouse. Unpaired Student’s *t* test, *=p < 0.05;

**Figure S8**

**
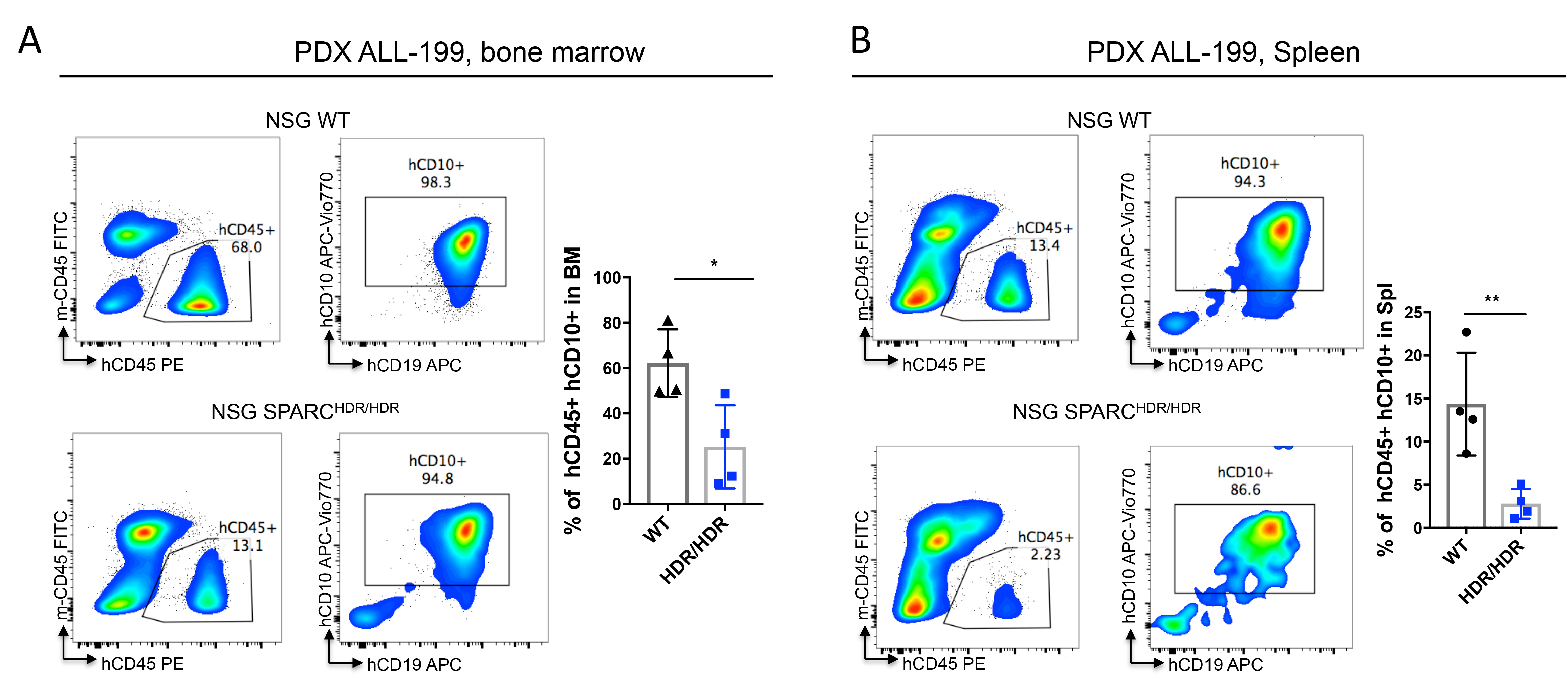
**

**Supplemental figure 8** Representative gating schemes and percentage of human CD45^+^ CD10+ cells in the bone marrow **(a)** and spleen **(b)** of WT (n=4) and SPARC^HDR/HDR^ (n=4) NSG mice engrafted with 3.10^5^ cells from a BCP-ALL relapsed case (ALL-199). Mice were analyzed 7 weeks post transplant. The data show a significantly reduced leukemic burden in both spleen and BM of SPARC^HDR/HDR^ mice. Each dot represents an independent mouse. Unpaired Student’s *t* test, *=p < 0.05; **=p<0.01
